# Exploring the impact of chia seeds and matcha green tea on gene expression related to the puberty pathway in growing male New Zealand white rabbits

**DOI:** 10.1007/s11250-025-04391-x

**Published:** 2025-04-02

**Authors:** Ahmed S. H. Soliman, Shymaa Sobhy Mourad, Amira Abdalla Abdelshafy Mohamed

**Affiliations:** 1 Department of Animal Production, Faculty of Agriculture, New Valley University, Al Kharga City, New Valley Egypt; 2 Immunology and Hematology Division, Department of Zoology, Faculty of Science, Arish University, Al-Arish, North- Sinai 45511 Egypt; 3 Department of Animal Production, Faculty of Environmental Agricultural Sciences, Arish University, Al-Arish, North- Sinai 45511 Egypt; 4 College of Veterinary Medicine, Northwest Agriculture and Forestry University, Yangling, 712100 China

**Keywords:** Chia seeds, Matcha tea, Growing rabbits, Gene expression

## Abstract

Abundant direct and in-direct genes are involved in regulating sexual hormones, and reproductive process under nitrite antioxidant plants in rabbit feed. However, there is not enough information about the role of chia seeds and matcha tea as anti-oxidative plants inhibit some direct and in-direct genes related to puberty of growing male rabbits. In this study New-Zealand White (NZW) rabbits treated with chia seeds and matcha tea in water from age after weaning to marketing age about two months ago and determent some sexual hormones, direct and in-direct genes related to puberty and reproduction process. Our data showed total testosterone measured by ELISA increased significantly in chia rabbits compared to control. ELISA analysis revealed that there were no alterations in the levels of follicle stimulating hormone (FSH) and luteinizing hormone (LH) in the treated groups. Direct genes such as doublesex and mab-3 related transcription factor 1 (DMRT1), sex-determining region Y protein (SRY), and gonadotropin-releasing hormone 1 (GnRH1) determent by qPCR show up-regulating in matcha groups comparable to control group. While in-direct genes follicle stimulating hormone receptor (FSHR) and estrogen receptor 1 (ESR1) detected by qPCR showed up-regulated in matcha rabbits compared to control rabbits. But, luteinizing hormone receptor (LHR) gene was down-regulated in matcha group, and it was up-regulated in chia seeds groups. The prolactin receptor (PRLR) gene investigates down-regulation in all treatment groups. Collectively, matcha tea as one of antioxidant plants were involved those genes studied and activated via hypothalamic- pituitary gonadal axis and led to early puberty in growing male NZW rabbits.

## Introduction

In Egypt, rabbit production is considered one of the most important projects that could contribute significantly to solving the problem of meat shortage. Therefore, there is an expansion in this industry, especially in tropical and subtropical regions. Both assisted reproductive techniques and pubertal onset are regulated by genetic, nutritional, and environmental factors (Mancini et al. 2022). Genetic, nutritional, and environmental factors intersect to regulate the complex mechanisms involved in assisted reproductive techniques and the initiation of puberty, influencing both the timing and results. The onset of puberty signifies the emergence of secondary sexual traits and reproductive capability, a process initiated by a blend of genetic, nutritional, and environmental influences (Manotas et al. 2022). Nutrition has a relationship between physiological parameters, psychological changes, sexual maturation, and fertility in male/female humans and animal husbandry (Folley 1949). A significant portion of reproduction status is determined by genotype, specifically, and phenotype as well as environmental interactions (GxE). Some environmental interactions such as nutrition and climate were important for achieving environmental balance (Chuma-Alvarez et al. 2021).

Different nutrition supplements in human and animal food during growth periods improve their own health and puberty characteristics (Chuma-Alvarez et al. 2021). Many supplements such as medicinal plants, herbs, minerals component, growth elements, and antioxidant plants can lead to positive effects on fertility in male and female animals (Menezo et al. 2016). Some direct and indirect genes can affect male animal fertility, such as DMRT1 (doublesex and mab3 related transcription factor 1), SHY, and GnRH1 (Sex Hormone important for reproduction) as direct genes and FSHR, LHB, ESR1, and PRLP as indirect genes.

DMRT1 gene is necessary for preserving the sertoli cell phenotype in post-natal mammalian testis and is essential and appropriate for testicular differentiation in male mice even in adults (Ge et al. 2017; Matson et al. 2011) and playing regulatory networks safeguard gonadal sex long which the fetal choice between male and female (Loffler et al. 2003; Raymond et al. 1999). The SRY gene functions in males play as a switch from undifferentiated gonadal somatic cells to testis development and plays many key roles in this process. These constitute the SRY gene transcriptional regulation network, the additive action of which governs testis determination and is controlled by a single gene on the Y chromosome (Merone et al. 2017). The GnRH1 gene is neuropeptides and mutations linked by hypogonadotropic, GnRH1 presented in testicular tissue of fetal, mature rats and adult humans was found positively (11,12). The physiological role of GnRH1 still unclear but, previous study reported that GnRH1 of testicular spend anti-steroidogenic impact and inhibiting testosterone production (Franssen and Tena-Sempere 2018).However, recent study GnRH1 expressed in ley dig cell in mammalian and showed up-regulated testosterone level through a post-receptor PGF2α pathway (Zhang et al. 2024).

Although FSHR, LHB, ESR1, and PRLP genes are common in the female body, they also have an indirect role in males. Those genes established in both sexes display a rapid onset of adult maternal care (Moran et al. 2020). PRLP plays as a pro-survival for spermatozoa by suppressing sperms capacitation via inhibition of SRC kinase, a family of protein tyrosine kinases, and activation and stimulation of AKT, a serine/threonine-specific kinase domain, and/or a C-terminal regulatory domain in male mammalian (Moran et al. 2020).

Additionally, the effects of antioxidant plants such as chia seeds and matcha green tea on fertility hormone secretion and fertility-related gene pathways in male animals have not been reported. In this study aims to investigate how the expression of these genes and fertility-related hormones is affected in the body and testicular tissue of rabbits following the use of antioxidant plants. In this context, there is a shortage of information about the potential of using chia seeds and matcha green tea as feed additive for livestock, particularly rabbits. The main objective of this study is to explore the potential of chia seeds and matcha green tea as a feed additive and its effect on fertility parameters through fertility hormones and gene pathways in rabbits, serving as a model for studying testicular function.

## Material and methods

### Ethics statements

The animal involved in this study underwent evaluation and approval by the Research Ethics Committee of Arish University, Egypt. The Ethical Committee protocol number is provided: (ARU/Agri.21).

### Experimental design

The present study was conducted at Rabbit Production Experimental Farm and Research, Faculty of Environmental Agricultural Sciences, Arish University, Egypt during 25/9/2023 and 25/11/ 2023. After being weaned at 35 day of age, eighteen male New-Zealand white rabbits were randomly separated into three groups (each group had six rabbits) according to their weight (505.55 ± 6.14 g). All rabbits were provided with commercial pelletized rabbit feed ad-libitum as basal feed. The experimental groups were as follows; control: a basal diet and tap water without supplementation, chia group: basal diet + chia seeds powder added as 0.075 gm/3-L tank water for three rabbits, and matcha group: basal diet + green tea light powder added as 0.037gm/3-L tank of water for three rabbits. The treated water was changed twice per week. A total of 3 rabbits were housed in a galvanized wire cage (60 cm × 40 cm × 30 cm) with a feeder and an automatic nipple drinker linked with tank. All rabbits were kept in the same warehouse, time, management, hygiene, and environmental case over the experimental duration. The average temperature was 27.50 ◦C in closed ambient and the daily photoperiod was a 14:10 h light–dark cycle with a semi-continuous lighting program. The fundamental experimental diet was formulated to meet the nutrient requirements of rabbits, according to NRC (NRC 1977). The experimental processes confirmed 10 weeks from the 5th to the 15th week of the rabbits’ age. The contents of the commercial pelletized rabbit feed were 15% yellow corn, 15% barley, 16% wheat bran, 18% soybean meal, 35% alfalfa hay, 3% molasses, 0.6 di-calcium phosphate, 0.6% limestone, 0.5% NaCl and 0.3% vitamins and minerals premix. The chemical analysis of the basal diet according to AOAC (2010) and shown in Table 1.

**Table 1:** Chemical composition of CFM (% DM basis)

Item	DM	OM	CP	EE	CF	NFE	Ash
CFM	90.2	92.61	18.72	3.02	13.85	57.02	7.39

*CFM* Concentrate Feed Mixture, *DM* Dry Matter, *OM* Organic Matter, *CP* Crude Protein, *CF* Crude Fiber, *NFE* Nitrogen Free Extract

### Collect samples

At the end of the experimental period, all experimental animals were slaughtered after fasting for 12 h; the blood samples were collected from the slaughtered rabbits in clean anticoagulant tube containing EDTA. Blood samples centrifuged at 3000 × for 15 min at 6ºC. Plasma samples were harvested into sterile tubes and stored at −20°c until used. And collected testis tissue and stored it at −80 for further experiments.

### Enzyme-linked immunosorbent assay (ELISA)

Different endocrinology sexual units such as Total Testosterone Hormone, and Follicle stimulating hormone (FSH) and Luteinizing Hormone (LH), were measured by ELISA *Sysmex* kits were done on *Sysmex (Sysmex, Japan)* in blood plasma and used following the manufacturer’s protocols.

### Total mRNA extraction and real-time quantitative PCR

Total RNA was extracted from tissues using TRIzol, and complementary DNA was synthesized according to the manufacturer’s instructions using a reverse transcription kit (Qiagen, Germany). Real-time PCR was performed using SYBR Premix Ex TaqTM (Moran et al. 2020). The sequences of specific primers were applied to extend the related 7 genes that are listed in Table 2. Glyceraldehyde 3-phosphate dehydrogenase was applied as a control gene (Joseph 1994). The messenger RNA (mRNA) quantification was analysis using the fold change 2^-ΔΔCT method (Winters et al. 2014), this experiment was performed in triplicate. The relative quantity of each sample was normalized to the level of the housekeeping gene.

**Table 2:** The sequences of primers used to real-time PCR

Gene	Primer	Accession number
DMRT1	AF: 5, -GAGGGTGATTTCTGGGATCTTG-3^,^ AR: 5, -CTCAGAAGCGCCATCCTATTC-3^,^	XM_008265225.3
ESTROGEN	AF: 5, -GAGGGTGATTTCTGGGATCTTG-3^,^ AR: 5, -CTCAGAAGCGCCATCCTATTC-3^,^	XM_008263695.2
FSHR	AF: 5, -GAGGGTGATTTCTGGGATCTTG-3^,^ AR: 5, -CTCAGAAGCGCCATCCTATTC-3^,^	XM_002709718.2
LHR	AF: 5, -GAGGGTGATTTCTGGGATCTTG-3^,^ AR: 5, -CTCAGAAGCGCCATCCTATTC-3^,^	OL331955
GNH	AF: 5, -GAGGGTGATTTCTGGGATCTTG-3^,^ AR: 5, -CTCAGAAGCGCCATCCTATTC-3^,^	OP791887
SRY	AF: 5, -GAGGGTGATTTCTGGGATCTTG-3^,^ AR: 5, -CTCAGAAGCGCCATCCTATTC-3^,^	NP_898968.1
PRL	AF: 5, -GAGGGTGATTTCTGGGATCTTG-3^,^ AR: 5, -CTCAGAAGCGCCATCCTATTC-3^,^	NC_067385.1
GAPDH	AF: 5, -GAGGGTGATTTCTGGGATCTTG-3^,^ AR: 5, -CTCAGAAGCGCCATCCTATTC-3^,^	M32999

### Statistical analysis

Collected data were subjected to one-way analysis of variance (ANOVA) using SAS v.9.1. The differences between the means were determined by Duncan multiple comparison test (Duncan 1955). The model used was Y_ij_ = µ + A_j_ + £_ij_

$$Y_{ij}=Observationtraits,\mu=Overallmean,A_j=Experimentaltreatment,\;\pounds_{ij }=Randomerror$$

## Results

### Sexual hormones determined under the usage of chia seeds and matcha green tea

**Fig. 1:**
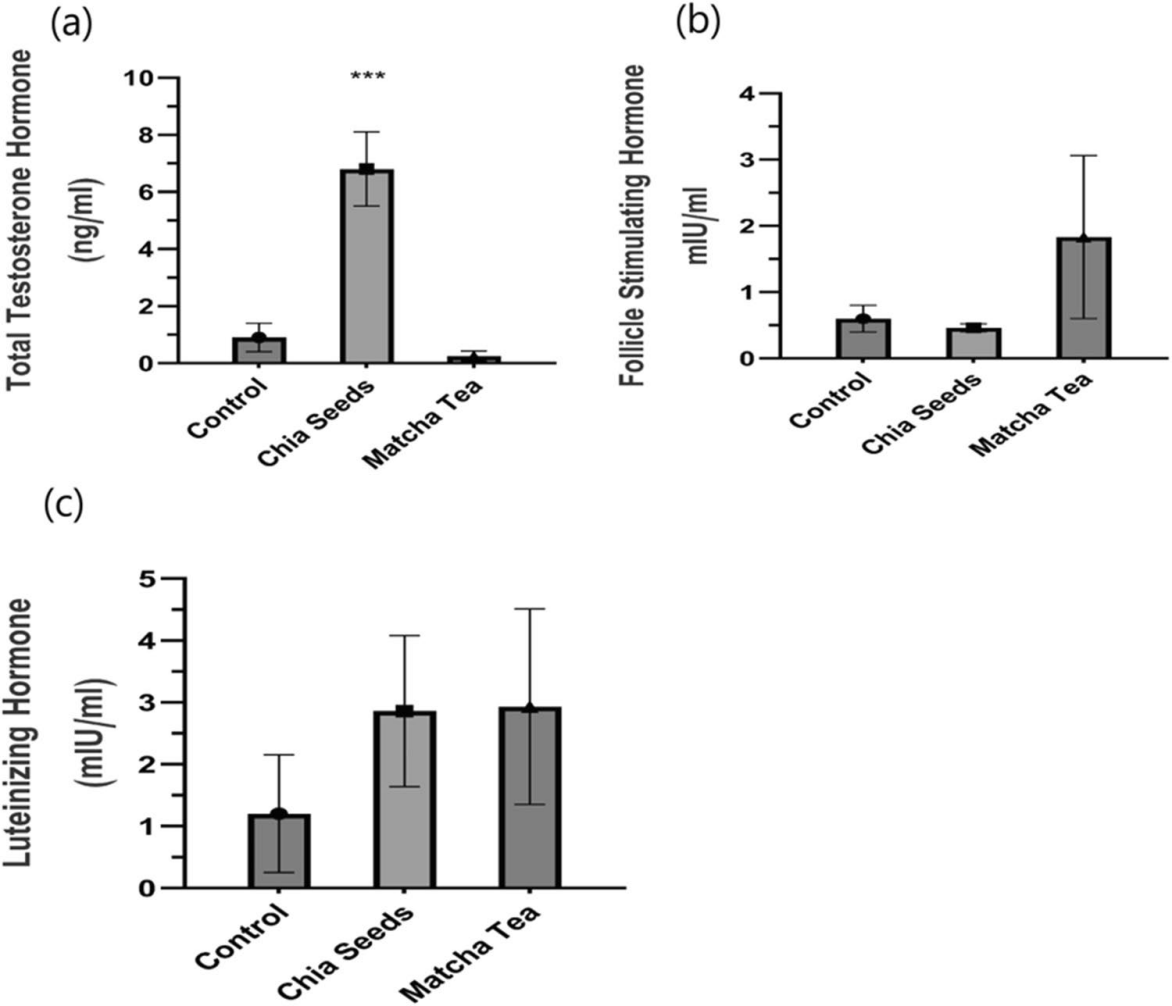
Sexual hormones measured by ELISA in blood plasma of growing male rabbits after treated with chia seeds and matcha tea. Total testosterone (1-a), Follicle stimulating Hormone (1-b). Luteinizing Hormone (1-c). Data are presented as means ± standard error of three independent experiments of six rabbits each. The asterisks show significant differences between all groups (^***^p < 0.005). ELISA: enzyme-linked immunosorbent assay

To investigate the concentrations of total testosterone hormone, follicle-stimulating hormone, and luteinizing hormone in plasma of New-Zealand White Rabbits after treated by chia seeds and matcha green tea Fig. 1, the results revealed a significant increase in total testosterone hormone in the chia seeds treatment group compared to the control group, while matcha green tea showed a significant difference compared to control group, as shown in Fig. 1a. In addition, our data in Fig. 1b, c showed follicle-stimulating hormone and luteinizing hormone levels were not affected by chia seeds and matcha tea compared to the control group.

### Several direct and indirect genes related to fertility are detected in testes tissues of New-Zealand white rabbits

#### Some direct genes as DMRT1, SRY, and GnRH1 regulate fertility in male rabbits

**Fig. 2:**
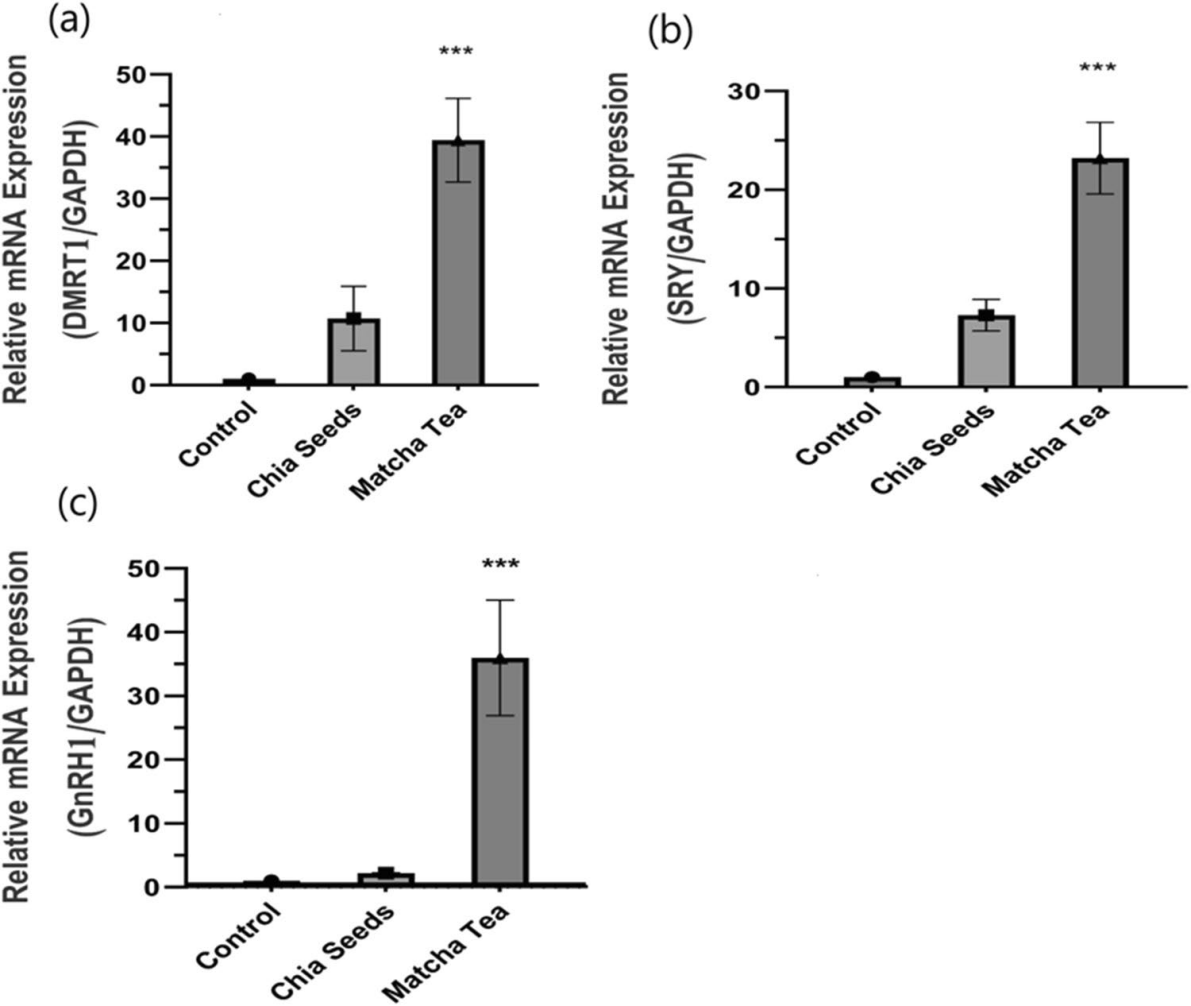
Direct genes measured by qPCR in testes tissue of growing male rabbits after treated with chia seeds and matcha tea. DMRT1 mRNA expression (1-a), SRY mRNA expression (1-b). GnRH1 mRNA expression (1-c). Data are presented as means ± standard error of three independent experiments of six rabbits each. The asterisks show significant differences between all groups (^***^p < 0.005). mRNA: messenger RNA; qPCR: quantitative PCR

To identify genes involved in the direct pathway of fertility in male rabbits, such as DMRT1, SRY, and GnRH1 genes shown in Fig. 2 and data investigated that depicting chia seed and matcha tea treatments. Our results showed that there is a significant increase in DMRT1 gene expression in the matcha tea treatment group compared to the control group, but in chia seeds DMRT1 gene expression there was no significant change compared to the control group Fig. (2-a).

SRY gene described as a direct pathway of fertility in Fig. 2b data showed up-regulated and high significance in the matcha tea treatment group compared to the zero-treatment group. While chia seed treatment did not show a significant change compared to the zero-treatment group. In Fig. 2c, the GnRH1 gene is presented, and data demonstrated that matcha treatment up-regulated significantly compared to control treatment when detecting the GnRH1 gene. There was no significant difference observed between chia seed treated and control groups.

#### Abundant in-direct genes such as FSHR, LHB, ESR1, and PRLP coordinate to fertility in growing NZW rabbits

**Fig. 3:**
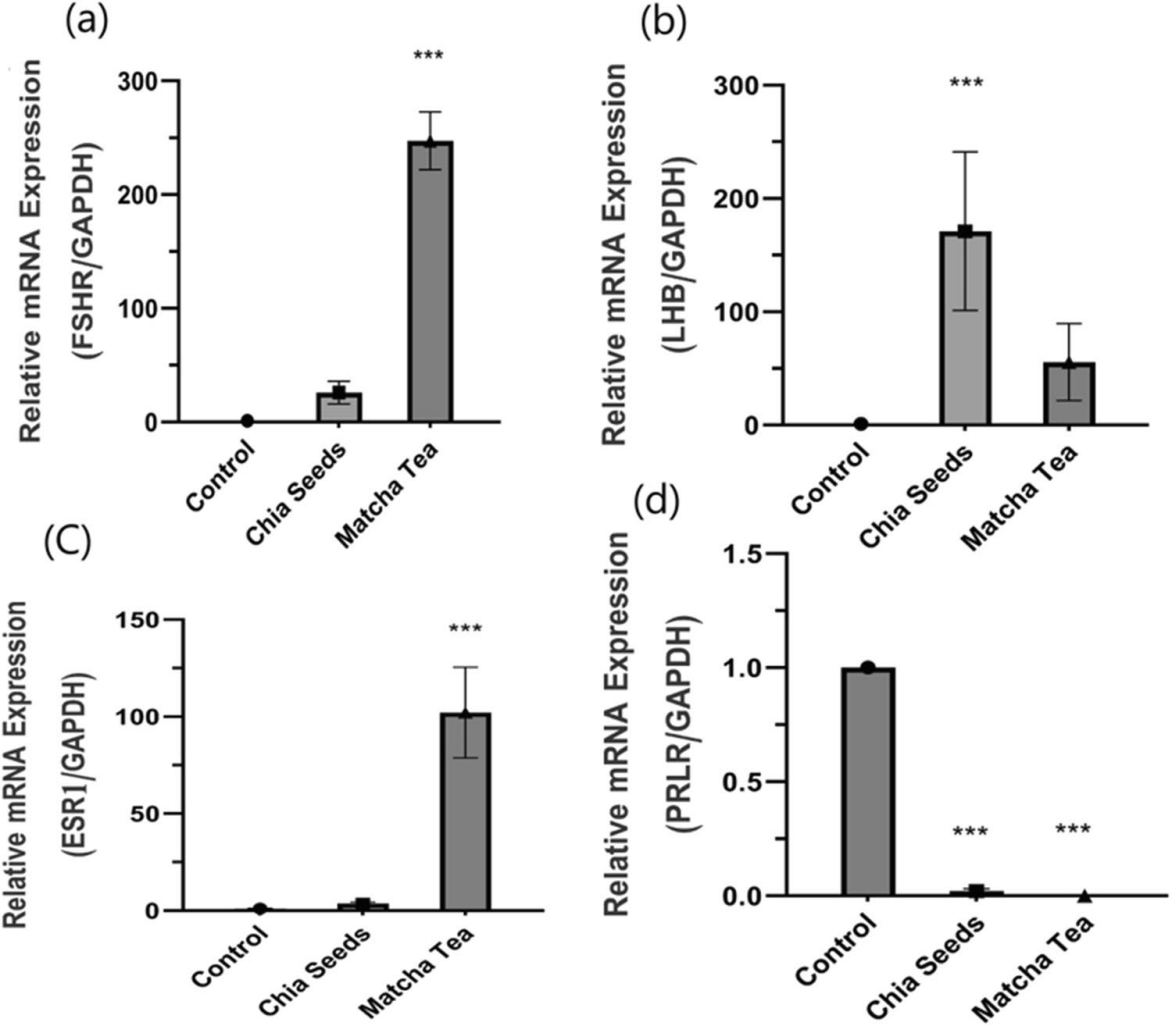
In-direct genes measured by qPCR in testes tissue of growing male rabbits after treated with chia seeds and matcha tea. FSHR mRNA expression (1-a), LHB mRNA expression (1-b). ESR1 mRNA expression (1-c). PRLR mRNA expression (1-d). Data are presented as means ± standard error of three independent experiments of six rabbits each. The asterisks show significant differences between all groups (^***^p < 0.005). mRNA: messenger RNA; qPCR: quantitative PCR

To investigate the effects of manipulated antioxidant plants feeding on puberty and fertility efficacy in growing NZW rabbits Fig. 3, the FSHR gene showed significant up-regulation in the matcha tea rabbits group compared to the control group. However, there was no significant difference observed between the chia seed group and the control group of rabbits, as shown in Fig. 3a. But in Fig. 3b the chia seed group exhibited a significantly higher expression of the LHB gene compared to the control group, whereas the matcha tea group did not show a significant change compared to the control group.

The ESR1 gene, presented in Fig. 3c, showed a significantly higher expression in the matcha group compared to the control group, while there was no significant change in the chia seeds group compared to the control group.

PRLR gene expression, depicted in Fig. 3d, evidenced a significant down-regulation (*p* < 0.001) in both treated groups (chia and matcha) compared to the control group Fig. 3d.

## Discussion

There have been few studies providing evidence that nutrition from chia seeds and matcha tea influences sexual hormones and gene expression related to puberty and fertility, especially in male NZW rabbits. There is limited information on the 24-h pattern of gonadotropin release, such as FSH or LH on possible sex-related differences in gonadotropin hormones, which are particularly important within the hypothalamic-pituitary–gonadal (HPG) axis of vertebrates or on prolactin (PRL) release of rabbit pups (Cano et al. 2005). In the current study, we demonstrate the production and validation of total testosterone hormone and gonadotropin hormones such as FSH and LH in growing rabbits under different antioxidant plant supplements. The data showed a significant increase in total testosterone hormone with chia seeds supplements compared to control, while there was no change in gonadotropin hormones in both treats. Our findings are consistent with the earlier reports (El-Speiy et al. 2023) who found that several supplements such as Alpine galangal and Zinc treats increased significant in sperm percentage, motility, and reproductive hormones such as testosterone, FSH, LH, E217β, and P4 in California rabbit bucks. Another study reported a decrease significantly in sperm count and motility percentage as well as a decrease in the testes weight, seminal vesicle, epididymis, and ventral prostate with long-term feeding of Osmium sanctum leaves as a kind of supplementation diet in adult albino rats (El-Speiy et al. 2023).

An explanation for this modality of changes in hormone levels could be that chia and matcha supplementation diets contain some androgenic elements, such as Omega3 found in chia seeds and Matcha tea components like thiamine, caffeine, chlorophyll, phenols, and diverse types of catechins (Kochman et al. 2020). These elements might sufficiently increase occurring testosterone levels to prohibit LH, However, not due to accumulation in the testis at the assessed concentration for normal spermatogenesis. But the decreased LH levels will reduce intra-testicular production of testosterone level by Leydig cells. These results miniature levels of spermatogenesis those explains the relationship of hypogonadism symptoms with the levels of sex hormones in males and it where normal balance in these hormone levels can have substantial effects on male reproduction (Sethi et al. 2010).

Several direct genes such as DMRT1, SRY, and GnRH1 showed significantly doubled expression in matcha-treated groups compared to the control in this study. Our results exposed that chia seeds increased the expression of DMRT1 and SRY genes but did not change significantly compared to control group. The involvement of DMRT1 in vertebrate testes differentiation, while SRY is the testes-detecting gene, remains absent as reported by (Dujardin et al. 2023) and SRY expression gene increased during the XY- chromosome is developing in testes. In male rabbits, researchers found that DMRT1 gene kept its leadership in sex definition, where SRY is responsible for testis determination (Song et al. 2017). However, DMRT1 might be demanded for chromatin reset on the enhancer 13 location to qualify SRY binding and SOX9 gene expression since the starting of testes differentiation. In the mouse, that promote more rapidly, DMRT1 could no longer be important for SRY gene role as the chromatin statement of the fetal supporting cells would be more permissive (Raymond et al. 2000).

GnRH1 is one of neuropeptides Y cerebrated to be one of the key regulators of hypothalamic-pituitary–gonadal axis in vertebrates. Recent study reported that the decrease of gonadotropin secretion led to partial lack of pubertal development in mammalian that case causes by congenital hypogonadotropic hypogonadism (Bouligand et al. 2010) and GnRH1 gene validating definitively the vital function in mammalian pubertal development and reproduction process. Our data disagreement in some studies reports above, in this study, GnRH1 gene significantly up-regulated expression in matcha treats, chia treats showed increased expression while there is not significantly observes comparable to control. In my interpretation, GnRH1 acted as the overseer of the pituitary gland, orchestrating the regulation of sexual genes and hormones within specific groups. This orchestration potentially contributed to the onset of early puberty and the progression of reproductive development.

Furthermore, FSHR, LHB, ESR1, and PRLP coordinate fertility in growing NZW rabbits. In the present study, data investigated that FSHR gene up-regulated significance in matcha treated groups, while the LHB gene exhibited significantly up-regulated in chia groups compared to control as shown in Fig. 3a, b. in addition, we clarified this data above and noted that it led to the physiological and normal functioning of sexual hormone functioning. FSHR is involved in Sertoli cell proliferation, spermatogenesis, and germ cell survival in the testis, and the FSH signal transduction pathway via FSHR plays a crucial role in regulating the spermatogenesis in vertebrate’s testes (Bhartiya and Patel 2021; Collins et al. 2003).

The ESR1 gene in this study showed a significant increase in matcha groups compared to control. Our data agree with several studies that show ESR2 gene function in the male tract is still controversial in mice, some studies demonstrated normal male fertility with ESRs (Krege et al. 1998). This gene is produced in the adult testes of many mammalian gonads, and testicular estrogens could be involved in the spermatid transition- spermatocyte the testis rabbits, and in the acquisition of sperm motility in the epididymis and absence of testicular Estrogen led to sperm defects (Dewaele et al. 2022).

On the other hand, the PRLP gene showed significantly down-regulated expression in both chia and matcha groups compared to the control. Some studies have reported that PRL might promote the function of the prostate, testes, and reproductive accessory tissues, and the reproductive process of male animals through its unclear roles and indirect involvement in the reproductive system of male mammals (Cao et al. 2015; Goffin et al. 2011).

## Conclusions

It can be concluded that matcha green emerged as the most effective among all groups. Puberty and fertility may be participating to species-specific differences in the way genes respond to hormonal or environmental stimuli, such as nutritional status, in different tissues. In this study, all genes related to puberty and fertility were affected by matcha green tea as a dietary supplement in growing NZW rabbits.

## Data Availability

All data generated or analyzed during this study are included in this published article.
